# Critical Overview of Screening Tools for Detecting Bipolar Disorders

**DOI:** 10.62641/aep.v53i5.1924

**Published:** 2025-10-05

**Authors:** Micaela Dines, Carolina Hernandorena, Veronica Grasso, Gustavo Vazquez

**Affiliations:** ^1^Seccion de Salud Mental, Hospital General de Agudos Dr. Juan A. Fernández, C1425AGP Ciudad Autónoma de Buenos Aires, Argentina; ^2^Instituto de Neurociencia Cognitiva y Traslacional (Consejo Nacional de Investigaciones Científicas y Técnicas - Fundación INECO - Universidad Favaloro), C1060AAF Ciudad de Buenos Aires, Argentina; ^3^Centre for Neuroscience Studies, Queen’s University, Kingston, ON K7L3X4, Canada; ^4^Centro Integral de Psicoterapias Contextuales (Fundación CIPCO), X5000KBB Córdoba, Argentina; ^5^Department of Psychiatry, School of Medicine, Queen’s University, Kingston, ON K7L3X4, Canada; ^6^International Consortium for Mood & Psychotic Disorder Research, McLean Hospital, Belmont, MA 02478, USA; ^7^Departamento de Neurociencias, Universidad de Palermo, C1186AAN Buenos Aires, Argentina

**Keywords:** bipolar disorders, diagnosis, screening instruments, sensitivity, specificity

## Abstract

This overview aims to explore the key screening tools for detecting bipolar disorders (BDs): the Mood Disorder Questionnaire (MDQ), Bipolar Spectrum Diagnostic Scale (BSDS), Hypomania Checklist (HCL-32), and Rapid Mood Screener (RMS), while offering guidance to healthcare professionals in selecting the most appropriate tool for each clinical scenario. The MDQ is widely utilized due to its high specificity (0.90) for identifying Bipolar Disorder (BD) in psychiatric consultations, although it is more sensitive to bipolar I than bipolar II. The BSDS, designed to encompass a wider range of bipolar spectrum symptoms, exhibits a sensitivity of 0.70 and specificity of 0.89, which makes it a complementary tool to the MDQ. The HCL-32 concentrates on detecting hypomanic traits in Major Depressive Disorder (MDD) patients, showing good sensitivity (80%) but lower specificity (51%). It is particularly effective for distinguishing BD from unipolar depression, although it cannot differentiate between Bipolar Disorder type I (BDI) and Bipolar Disorder type II (BDII). The RMS is a newer tool that quickly screens for manic symptoms and risk factors, boasting a sensitivity of 0.88 and a specificity of 0.80. Together, these screening instruments facilitate the early identification of BDs, though positive results should always be followed by a thorough clinical evaluation. Employing multiple tools simultaneously can improve diagnostic accuracy and more effectively capture the diverse presentations of BDs.

## Introduction

Bipolar disorders (BDs) are severe medical conditions that significantly impact the quality of life of those affected. Their influence on morbidity and mortality is evident not only in suicidal behaviour but also in decreased life expectancy, mainly due to early onset, chronicity, and economic burden [1, 2, 3]. Moreover, while the global prevalence is estimated at 1% for all forms of BDs, it rises to 4.4% of the population[1]. It is important to note that the Global Burden of Disease Study attributes 9.9 million years of disability-adjusted life years (DALYs) to BDs, primarily during their depressive phase, making them the sixteenth leading cause of disability among individuals aged 10 to 24 worldwide [4]. Currently, the diagnosis of BDs remains strictly clinical, which poses a challenge for healthcare professionals. This challenge arises from multiple factors, including the lack of supplementary studies, the stigma associated with mental health disorders, patients’ hesitation to report hypomanic or manic symptoms [5], and, fundamentally, the fact that the disorder often begins with a depressive episode, leading to misdiagnosis as other conditions [4, 5]. The primary differential diagnosis in cases of BDs is Major Depressive Disorder (MDD). The importance of making an accurate diagnosis lies not only in providing effective treatment but also in preventing the use of antidepressants in patients with BD, as this may result in increased mood instability and a greater risk of triggering a manic or hypomanic episode. For this reason, the use of antidepressants is generally discouraged in patients with this diagnosis. Furthermore, the use of antidepressants has been associated with chronic irritability (also known as Chronic Irritable Dysphoria or ACID), rapid cycling, mixed symptoms, disturbances in circadian rhythm, and treatment resistance [6]. It is crucial to emphasize that no additional studies currently support clinical diagnosis. Consequently, there remain gaps in the ability to identify the disorder, with patients typically taking an average of 8 to 10 years to obtain an accurate diagnosis [1, 4]. As a result of this diagnostic delay, patients are subjected to ineffective or harmful treatments, as well as an increased risk of suicide. Moreover, from a public health standpoint, these patients incur higher costs due to receiving ineffective treatments, which leads to greater hospitalization rates and inappropriate use of therapies [7]. It is essential to emphasize that routine screening for depression is recommended at the primary care level, while screening for BD is not. Since depression is the leading cause of morbidity in BD and most patients seek care during this phase of their illness, the lack of screening at this stage is believed to contribute to diagnostic delays [8]. In this context, screening tools are vital as they provide standardized resources that assist clinicians in identifying, quantifying, and monitoring symptoms over time, thereby improving diagnostic accuracy and treatment planning. The use of standardized scales allows healthcare professionals to perform a systematic evaluation of bipolar symptoms and facilitates the differentiation between manic, hypomanic, and depressive episodes. Among the most widely used tools in clinical practice are the Bipolar Spectrum Diagnostic Scale (BSDS), the Mood Disorder Questionnaire (MDQ), the Hypomania Checklist-32 (HCL-32) and newer tools such as the Rapid Mood Screener (RMS). Each scale possesses unique characteristics, strengths, and limitations, rendering them more or less suitable based on the clinical context and target population. The aim of this article is to explore the characteristics and applications of these scales in detecting BD, analyzing their advantages and limitations in clinical practice. This comparative analysis seeks to guide healthcare professionals in selecting the most suitable tool for each clinical situation, ultimately enhancing their understanding and management of this complex disorder. The particular features, pros, and cons of each tool are summarized in Table 1.

**Table 1.** **Comparison between screening tools for the detection of bipolar disorders**.

Scale	Type of test	Assessment	Function	Sensitivity	Specificity	Clinical context	Features
HCL-32	Screening	Self-assessment	Distinguish BD from MDD	80%	51%	General psychiatric consult	Advantages
						Specialized psychiatric consult Tested in inpatients and outpatients	Patients diagnosed with (BD and MDD), but also as a screening tool in general psychiatric practice
							Wide range of regions and cultural contexts
							Limitations
							Comorbid conditions increase the HCL-32 score
RMS	Screening	Self-assessment	Detecting possible cases of BDI	88%	80%	Primary care	Advantages
						General psychiatric consult	BDI screening
						Tested in outpatients	Primary care setting
							RMS-C also BDII
							Limitations
							Not for a more comprehensive assessment of the BDI
BSDS	Screening	Self-assessment	BDI, BDII, BDNOS	76% (BDI, BDII, BDNOS)	85%–93% (BDI, BD II, BDNOS)	Primary care	Advantages
				75% (BDI)		General psychiatric consult Tested in outpatient and inpatients	Detection of milder forms of BD, BDII and BDNOS
				79% BDII, BDNOS			Limitations
							Performance would vary depending on cultural and clinical context
MDQ	Screening	Self-assessment	BDI, BDII	73–80%	90%	Primary care	Advantages
	Tracking symptoms					General psychiatric consult	Identify BD
	Research					Tested in outpatients and inpatients	Short and simple application (5 minutes)
							Use in primary care, initial screening measure
							Limitations
							Possible inaccurate responses
							Lower sensitivity for detecting BDII
							Difficulty in distinguishing BD from other psychiatric conditions with overlapping symptoms

MDQ, Mood Disorder Questionnaire; BSDS, Bipolar Spectrum Diagnostic Scale; HCL-32, Hypomania Checklist; RMS, Rapid Mood Screener; BDI, Bipolar Disorder type I; BDII, Bipolar Disorder type II; MDD, Major Depressive Disorder; BD, Bipolar Disorder; RMS-C, Rapid Mood Screener-Chinese Version; BDNOS, Bipolar Disorder Not Otherwise Specifed.

## Mood Disorder Questionnaire

The MDQ is a widely used self-report screening tool designed to detect BD. Developed by Hirschfeld and colleagues in 2000, it provides a quick and efficient way to identify individuals at risk for BD, particularly Bipolar Disorder type I (BDI). As a screening tool, it aims to identify potential cases of BD, which clinicians should further investigate to confirm or rule out the positive screening result.

### Structure

The MDQ comprises 13 items that evaluate the presence of manic or hypomanic symptoms, followed by questions regarding the temporal co-occurrence of these symptoms and their impact on daily functioning. Patients are asked to indicate whether they have ever experienced these symptoms and whether they occurred together, followed by an assessment of the resulting functional impairment [9]. Reported sensitivity ranges from 0.70 to 0.90 and specificity from 0.70 to 0.90, depending on the study and the population assessed Its positive predictive value (PPV) and negative predictive value (NPV) have been tested in both inpatient and outpatient populations, ranging from 47% to 63% for PPV and from 78% to 86% for NPV. Overall, the MDQ is regarded as a valid and reliable screening tool for BD, particularly when used alongside clinical interviews and other diagnostic measures [9].

### Validation and Performance Across Different Clinical Contexts

The MDQ is most commonly used in clinical settings, particularly in primary care, where patients may not have been formally diagnosed with BD. Its brevity (usually taking less than 5 minutes to complete) and simplicity make it a valuable tool for primary care providers who may be the first to recognize the signs of BD. The questionnaire’s broad application is supported by its use in both community samples and psychiatric settings, where it serves as an initial screening measure to guide further diagnostic evaluation [10]. Despite its advantages, the MDQ has significant limitations. One major concern is its reliance on self-reporting, which may result in inaccurate responses due to recall bias or a patient’s lack of insight into their manic or hypomanic episodes. Furthermore, the MDQ often exhibits lower sensitivity for detecting Bipolar Disorder type II (BDII), particularly in individuals with mild or subthreshold hypomanic symptoms, potentially leading to false negatives in this population [11]. Additionally, while the MDQ evaluates a wide range of manic symptoms, it does not encompass the full spectrum of mood disorders, which may limit its diagnostic utility in distinguishing BD from other mood disorders or psychiatric conditions with overlapping symptoms [12]. In conclusion, the MDQ is a valuable and efficient tool for screening BD, particularly in primary care and community settings. While its sensitivity and specificity make it an effective initial screening tool, clinicians should be mindful of its limitations, particularly its reliance on self-report, lower sensitivity for BDII, and inability to fully capture the complexities of bipolar spectrum disorders.

## Hypomania Checklist 

HCL-32 is a self-assessment tool designed to assist in diagnosing BDII or the BD spectrum in patients who meet the criteria for an MDD diagnosis. While some depressive symptoms have been described as indicative of BD [13], patients experiencing a depressive episode can be incorrectly diagnosed as having MDD. Developed by Angst and colleagues in 2005, the HCL-32 seeks to identify past hypomanic symptoms in individuals currently going through a depressive episode.

### Structure

The HCL-32 presents nine questions. In question three, the 32-item checklist displays statements that define what is considered a “high” state. It serves as a retrospective checklist, where the patient should select “yes” or “no” for each presented statement. The 32 statements are divided into two factors: “active/elated” and “irritable/risk taking”. The active/elated factor includes items 2 to 6, 10 to 13, 15, 16, 19, 20, 22, 24, and 28, while the irritable/risk taking factor comprises items 7, 8, 9, 21, 25, 26, 27, 31, and 32.

The scoring of the HCL-32 represents the number of positive responses to the 32-item checklist. As reported in its original publication a total score of 14 or more demonstrates a sensitivity of 80% and a specificity of 51% in distinguishing between BD and MDD. The PPV and NPV for this cut-off are 73% and 61%, respectively. Cronbach’s alpha for the total scale was 0.82 in the Italian sample and 0.86 in the Swedish sample.

### Validation and Performance Across Different Cultural and Clinical Contexts

The HCL-32 was initially conceptualized in German, then translated and first tested among Italian and Swedish populations, achieving the noted 80% sensitivity and 51% specificity [14]. Since then, the scale has been validated across multiple countries and languages. Its transcultural validity has been examined by Gamma *et al*. [15], who performed a measurement of invariance test to assess the HCL-32-R2’s ability—a modified version that includes gambling and eating habits—to consistently measure hypomania across different geographical regions or cultures. A total of 5606 subjects from five different regions (Iberia, Central Europe, Eastern Europe, North Africa/Near East, and Far East) were included. Only three items in the HCL-32-R2 displayed non-invariant primary factor loadings across cultures: reduced need for sleep, flight of ideas, and coffee consumption. These results are overall favorable towards HCL-32-R2, suggesting that its psychometric properties were mostly culture-independent.

The HCL-32 was primarily tested on participants diagnosed with affective disorders (BD and MDD), but it may also serve as a valuable screening tool in general psychiatric practice. A study by Meyer *et al*. [16] assessed both the HCL-32 and MDQ for sensitivity and specificity in a sample of outpatients with and without mood disorders, examining whether the presence of psychiatric comorbidities influenced the performance of the scales. When comparing bipolar to non-bipolar participants, the HCL-32 demonstrated a sensitivity of 88% and a specificity of 36%, while the MDQ exhibited lower sensitivity (80%) but higher specificity (64%). This study also investigated the potential impact of comorbid Axis I or Axis II diagnoses on the scoring of both scales. The findings suggested that the presence of comorbid conditions generally increased the scores on both the HCL-32 and MDQ, irrespective of a BD diagnosis, but did not significantly enhance the positive screening of BD. Finally, this study also considered the PPV and NPV of the HCL-32, obtaining 40% and 86% values, respectively.

Overall, the HCL-32 is a valuable tool for screening BD in patients experiencing a depressive episode across various regions and cultural contexts. As it is a self-assessment tool, it can be easily implemented in most psychiatric settings without the need for specific training. Like any screening tool, a positive result warrants further evaluation and clinical judgment and should not be used as a diagnostic scale.

## Bipolar Spectrum Diagnostic Scale 

Early detection of BDs, particularly in their mild or unclassified forms, has been a significant concern in psychiatric research. Mild forms of BD, such as BDII or Bipolar Disorder Not Otherwise Specified (BDNOS), are often more challenging to identify, which can lead to misdiagnosis or delayed diagnoses. Various diagnostic tools have been developed in this context to enhance sensitivity and accuracy in identifying these disorders. One of the most notable tools is the BSDS, which has demonstrated effectiveness in identifying more severe cases of BD and detecting more subtle variants within the bipolar spectrum, such as BDII and BDNOS. Originally designed to improve the detection of BDII, the BSDS is presented in a story format, comprising 19 sentences that describe various symptoms. Completing the scale takes approximately 10 minutes. The BSDS was initially developed by Dr. Ronald Pies, who, as a psychopharmacology consultant, recognized that many patients with “treatment-resistant depression” were actually presenting undiagnosed bipolar spectrum disorders. Drawing from this experience, Dr. Pies created a tool to detect not only severe cases of BD but also those that are less apparent, such as patients with brief episodes of elevated mood that do not meet the DSM-IV criteria for hypomania [17].

### Structure of the BSDS

The BSDS consists of two parts. The first part includes a paragraph of 19 sentences with a positive valence that describes various symptoms of BD. Each sentence is connected to a blank space that the patient must mark if they believe the statement reflects their experience. Each mark adds one point. The second part of the scale asks the patient to evaluate how accurately the narrative of the 19 items represents their own experience. Response options range from 0 to 6 points, with “This story fits me very well” receiving 6 points and “This story does not describe me at all” receiving 0 points. The total score can range from 0 to 25, categorizing patients into four groups based on the likelihood of having BD: a score between 20–25 indicates a high likelihood, 13–19 indicates a moderate likelihood, 7–12 indicates a low likelihood, and 0–6 indicates very low likelihood.

The study by Nassir Ghaemi *et al*. [17] found that the BSDS exhibits high sensitivity, accurately detecting BD in 76% of cases. This is particularly noteworthy for patients with BDI, BDII, and BDNOS. The scale’s effectiveness stems from its capability to capture subtle bipolar symptoms that are often overlooked in traditional diagnostic criteria. Regarding PPV and NPV, studies have reported 87% and 52%, respectively, encompassing inpatient and outpatient samples [12].

### Validation and Performance in Different Cultural and Clinical Contexts

The BSDS has proven to be an effective tool for detecting BDs across the spectrum, ranging from the most severe to the mildest forms. However, its performance varies depending on the cultural and clinical context in which it is used, as demonstrated by various studies in different populations.

An example of this is the study conducted by Vázquez *et al*. [18], which validated the Spanish version of the BSDS within a Spanish-speaking population. In this study, the scale’s sensitivity was 0.70, slightly lower than that reported in the original research, but it had a specificity of 0.89, highlighting the BSDS’s usefulness in various cultural contexts [18]. Furthermore, the results revealed that sensitivity could be adjusted by modifying the cut-off threshold: by lowering the threshold from 13 to 12 points, sensitivity increased to 0.76, although this came with a slight decrease in specificity to 0.81, thereby illustrating the need to balance true case detection while minimizing false positives. In another study by Zaratiegui *et al*. [19] in Argentina, the MDQ and BSDS scales were compared in patients with mood disorders. The results indicated that both scales had comparable performance, although the MDQ exhibited slightly lower sensitivity and higher specificity for BDs compared to the BSDS. In this study, the specificity of the MDQ was 0.97, one of the highest reported, while the sensitivity of the BSDS was 0.67, consistent with previous studies that found sensitivities between 0.57 and 0.76 [17, 18]. Meanwhile, the specificity of the BSDS was 0.81, aligning with other studies where values ranged between 0.72 and 0.89. Zaratiegui’s study [19] also noted that the sensitivity of the MDQ was higher for BDI, at 0.70, compared to BDII (0.52) and BDNOS (0.31), consistent with patterns found in previous research. However, the MDQ was generally more effective at detecting BDs, while the BSDS was more sensitive to mild forms of BD, a finding also reported in earlier research [17, 18].

This pattern suggests that, while the MDQ is more effective at detecting more overt BDs, the BSDS is particularly useful for identifying milder forms of BD. This is important, as BDI is more easily recognized than bipolar spectrum disorders. In this context, self-report questionnaires such as the MDQ and the BSDS may be especially helpful for identifying milder BDs [19].

Supporting the effectiveness of the BSDS, an additional study by Zimmerman *et al*. [20] involving a sample of psychiatric outpatients found sensitivities of 75% for BDI and 79% for BDII/BDNOS, reinforcing the notion that the BSDS is especially effective at detecting milder forms of BD.

In conclusion, the results of these studies highlight the importance of considering the cultural and clinical context when applying the BSDS and the MDQ. Both questionnaires can enhance each other’s effectiveness in improving diagnostic accuracy, particularly in identifying milder forms of BD. Although further research is necessary to validate their effectiveness in diverse populations, the BSDS represents a significant advancement in the diagnosis and treatment of BD. Given its remarkable sensitivity to milder forms of BD, the BSDS could serve as a valuable tool in primary care for further investigating a potential BD diagnosis in individuals who screen positive. Its combination with other tools, such as the MDQ, could further optimize the detection and treatment outcomes for this disorder.

## Rapid Mood Screener 

The RMS is a self-assessment scale originally developed by Roger McIntyre for BDI in 2020. The questions relate to clinical features identified as predictors of bipolarity, including mood switching induced by antidepressants, early onset age, and the presence of hypomanic symptoms. It has an estimated sensitivity of 88%, specificity of 80%, an accuracy of 84%, a PPV of 80%, and a NPV of 88% [21].

### Structure

In its original English version, the patient must answer “YES” to 4 of the 6 total questions for the screening to be deemed positive. These questions aim to identify symptoms of the manic spectrum in patients with mood disorders and are regarded as the most discriminative: number of prior depressive episodes, comorbidities, age of onset, family history, treatment response, and manic symptoms—three items screening for bipolar I disorder risk factors and three that assess manic symptoms [21].

### Validation and Performance Across Clinical and Cultural Context

In addition to its original version, it has also been validated for BDII in its Chinese version by Yuhua Liao and colleagues Rapid Mood Screener-Chinese Version (RMS-C). The authors of this review are currently validating the Spanish version. Alongside studies assessing the scale’s sensitivity and specificity, which highlight its significance in primary care settings, Thase and colleagues [8] conducted a national survey in 2023 to gather the opinions of 200 healthcare professionals, including both primary care providers and psychiatrists, about the use of the RMS and its comparison with the MDQ. The results indicated that 84% of psychiatrists reported that the RMS would positively influence their practice, and 81% stated they would use the RMS prior to the MDQ (81% vs. 19%; *p* < 0.05) . The majority of both primary care professionals and psychiatrists reported that if given the choice between the RMS and the MDQ, they would prefer the RMS due to its essential attributes, such as brevity, ease of response, appropriate sensitivity and specificity, and its accessible scoring method

Screening for BDI in patients with depression represents a sensible and practical strategy for primary care providers to identify when a more comprehensive evaluation for BDI is warranted. The RMS was developed as a reliable and effective tool for screening bipolar I disorder, employing carefully crafted questions that use clear and precise language.

In conclusion, the RMS is an effective new screening tool for BDI, designed to assist clinicians in quickly identifying patients with depressive symptoms who may have BDI instead of MDD. Developed with both practicality and accuracy in mind, the RMS includes a limited set of straightforward, patient-friendly questions to lessen respondent burden and enhance its usability in fast-paced clinical settings. When compared to the MDQ within the same analysis group, the RMS exhibited superior test performance, requiring 60% fewer items [21].

## Discussion

The four scales included in this study are self-assessment tools. There are clear advantages to using self-rating tools; namely, they are time-efficient for both patients and healthcare professionals, as they require no training and can be easily scored [22]. However, potential biases may arise when implementing self-rating scales, as conscious or unconscious tendencies may distort responses (such as tendencies to exaggerate or conceal symptoms). Additionally, positive response bias and social desirability effects can also skew responses in self-rating scales [23]. Despite this, self-rating tools still hold value for screening in psychiatry, as they are intended to be used under clinician supervision rather than as diagnostic tools. They can provide insights into a patient’s symptoms and prompt further questioning during both primary care and psychiatric interviews. It is also important to consider methods for improving the reliability of self-ratings, such as Cronbach’s alpha. The included scales have strong evidence for internal consistency, such as HCL-32, which typically has a value greater than 0.80 in most reports, and similar results are observed for MDQ and BSDS.

Each tool offers benefits in various clinical contexts, including primary care, psychiatric settings, and research studies. Except for the RMS, all three screening tools have been tested in both inpatient and outpatient populations. The HCL-32, in particular, has primarily been tested within psychiatric consultations, demonstrating a higher PPV and lower NPV in the general psychiatry interview compared to mood-specific consultations. The MDQ and BSDS have also been tested in inpatient and outpatient settings, including primary care consultations, exhibiting the aforementioned PPV and NPV. The MDQ and RMS, due to their brief nature, are well-suited for initial screenings in busy settings, while the HCL-32 and BSDS provide deeper insights into hypomanic episodes and bipolar spectrum subtypes, making them valuable for specialized assessments. The MDQ can be applied in a general clinical context, both in psychiatric clinics and in primary care when there is suspicion of mood disorders, particularly if there is a history of manic or hypomanic episodes. It is useful for detecting more apparent cases of BD, such as BDI. In the context of the HCL-32, it is particularly useful for identifying symptoms of hypomania, particularly in patients with BDII or BDNOS, where hypomania is a central component. It focuses more on hypomanic episodes rather than full-blown manic episodes and is used when there is suspicion of BDII or a milder spectrum, particularly in patients presenting with elevated or irritable mood episodes that do not meet the criteria for mania but may impact daily functioning. Both the MDQ and HCL-32 can help determine the type of BD being faced. The RMS is a quicker and simpler tool, making it suitable for initial clinical consultations in primary care or psychiatric settings to provide a rapid overview of the patient’s mood-related symptoms. The BSDS is particularly helpful in identifying more subtle forms of BD, such as BDII, BDNOS, or even other less obvious presentations of BD. It is ideal for patients showing less clearly defined symptoms, such as episodes of elevated mood that do not fully meet the criteria for mania or hypomania. Its utility is crucial when BD is suspected in patients with subclinical episodes or when the presentation is insufficiently clear for a conventional diagnosis. It is also beneficial for patients exhibiting treatment-resistant depression symptoms, where a bipolar diagnosis has not been previously considered. However, few studies have been conducted in this area. In comparing tools, a meta-analysis conducted by Sayyah *et al*. [24] evaluated new tools such as the RMS and another scale named the *Bipolarity Index* (BI) against older scales like the BSDS, MDQ, and HCL-32. The authors concluded that the RMS was more precise (*p* < 0.0001) for detecting BDI than the other scales [24].

These instruments possess unique features and strengths, making them valuable in various clinical contexts. However, their combined use presents both advantages and challenges. On one hand, utilizing the different tools can enhance the sensitivity and/or specificity of the screening. While each tool targets distinct aspects of bipolar spectrum disorders, their collective application may capture a broader range of symptoms, including both manic and hypomanic episodes, along with mood fluctuations. For example, the MDQ and HCL-32 are particularly useful for identifying mania and hypomania. At the same time, the RMS is designed to assess rapid mood shifts, while the BSDS effectively detects subthreshold BD (e.g., cyclothymia or BDNOS). The BSDS is highly valued for its detection capabilities, although it does have limitations. While it effectively rules out a BD diagnosis, some studies suggest that it is not ideal for confirming the diagnosis due to its low positive predictive value. In this regard, the scale does not meet all the criteria for a formal BD diagnosis according to our current formal nosography (e.g., DSM or ICD), and its use in monitoring treatment progress is limited. Some studies suggest combining the BSDS with other tools, such as the MDQ, to improve diagnostic accuracy. This combined approach could represent an effective strategy for addressing the diverse presentations of BD in clinical practice [12]. On the other hand, using multiple tools increases the time commitment for both the patient and the clinician. The RMS is brief and straightforward, while the HCL-32 consists of 32 items, and the BSDS contains 18. Furthermore, few studies provide direct comparisons of screening tools administered within the same analysis population. Nonetheless, the combined use of these scales should be considered in both primary care and specialist consultations. Administering the MDQ or RMS alongside the BSDS may prove beneficial in primary care, as the MDQ and RMS are more likely to identify an underlying BDI diagnosis. In contrast, the BSDS has demonstrated greater sensitivity for milder presentations and BDII. Regarding the specialist psychiatric interview, using the HCL-32, which has shown significant sensitivity in identifying BDII, together with the MDQ or RMS could be especially advantageous.

## Conclusion

In conclusion, BDs are often under-recognized in various settings, and routine screening is advisable. Early assessment of BDs is crucial for effective treatment, as their diagnosis is frequently delayed due to several factors that contribute to increased morbidity and mortality associated with the disorder. In this context, employing screening tools enhances the detection of BDs. The implementation of screening tools should occur alongside a clinical evaluation, which includes gathering a medical history that assesses the patient’s background, such as previous hospitalizations or a family history of mood disorders. Furthermore, it may be more appropriate to use a scale tailored to the context of the interview, considering factors like the patient’s symptoms, the clinician’s experience, and the purpose of administering the scale. Generally, combining several scales or integrating these tools within a broader diagnostic framework may optimize the identification of BDs across different phases and improve clinical outcomes.

To complement the screening, hetero-applied, symptom-specific assessment scales for depression (HAM-D, MADRS) can be combined with those for mania/hypomania (YMRS). Although these scales are useful tools, an accurate diagnosis relies on a thorough assessment and continuous mood monitoring over time and during treatment.

Finally, it should be noted that most studies have been conducted in similar healthcare settings; therefore, further research in diverse clinical contexts is needed to strengthen the generalizability of these findings.

## Availability of Data and Materials

No datasets were generated or analyzed for this review. All information presented is derived from previously published studies cited in the reference list.
